# Technical Feasibility and Physiological Relevance of Hypoxic Cell Culture Models

**DOI:** 10.3389/fendo.2020.00057

**Published:** 2020-02-21

**Authors:** Jiri Pavlacky, Jan Polak

**Affiliations:** ^1^Department of Pathophysiology, Third Faculty of Medicine, Charles University, Prague, Czechia; ^2^Rare Diseases Research Unit, Department of Pediatrics and Adolescent Medicine, First Faculty of Medicine, Charles University, Prague, Czechia

**Keywords:** hypoxia, cell culture, animal model, *in vitro* model, pericellular oxygen, oxygen concentration, partial pressure, normoxia

## Abstract

Hypoxia is characterized as insufficient oxygen delivery to tissues and cells in the body and is prevalent in many human physiology processes and diseases. Thus, it is an attractive state to experimentally study to understand its inner mechanisms as well as to develop and test therapies against pathological conditions related to hypoxia. Animal models *in vivo* fail to recapitulate some of the key hallmarks of human physiology, which leads to human cell cultures; however, they are prone to bias, namely when pericellular oxygen concentration (partial pressure) does not respect oxygen dynamics *in vivo*. A search of the current literature on the topic revealed this was the case for many original studies pertaining to experimental models of hypoxia *in vitro*. Therefore, in this review, we present evidence mandating for the close control of oxygen levels in cell culture models of hypoxia. First, we discuss the basic physical laws required for understanding the oxygen dynamics *in vitro*, most notably the limited diffusion through a liquid medium that hampers the oxygenation of cells in conventional cultures. We then summarize up-to-date knowledge of techniques that help standardize the culture environment in a replicable fashion by increasing oxygen delivery to the cells and measuring pericellular levels. We also discuss how these tools may be applied to model both constant and intermittent hypoxia in a physiologically relevant manner, considering known values of partial pressure of tissue normoxia and hypoxia *in vivo*, compared to conventional cultures incubated at rigid oxygen pressure. Attention is given to the potential influence of three-dimensional tissue cultures and hypercapnia management on these models. Finally, we discuss the implications of these concepts for cell cultures, which try to emulate tissue normoxia, and conclude that the maintenance of precise oxygen levels is important in any cell culture setting.

## Introduction

Oxygen first began to significantly accumulate in the Earth's atmosphere with the advent of photosynthesis, a process enabling the ancestors of cyanobacteria to obtain hydrogen from water and combine it with atmospheric CO_2_ to produce hydrocarbon molecules (1, 2). Subsequently, most of contemporary life was presumably exterminated, having no line of defense against reactive oxygen species, in a process that has sometimes been labeled as the “oxygen holocaust.” However, conditions were ideal for the evolution of oxygen-consuming organisms who could take advantage of more energy-efficient aerobic metabolism. Oxygen thus became a necessary molecule that enabled the life of eukaryotic organisms including humans, because they acquire energy by oxidative phosphorylation where oxygen serves as the ultimate acceptor of electrons (3).

The universal oxygen demand in complex organisms created the requirement for an effective system that distributed oxygen into the entire body and satisfied the metabolic requirements of all tissues (3). Diffusion in the lungs and peripheral tissues is the key process in the transport of oxygen to the mitochondria; therefore, concentration gradients have developed across the human body leading to variable tissue O_2_ levels in different organs (4). Importantly, anaerobic metabolism has not been entirely forgotten by eukaryotic cells. In fact, some cells, such as erythrocytes, rely completely on anaerobic phosphorylation, whereas others resort to such means during diminished oxygen supply (i.e., hypoxia), for example, during intensive exercise (5, 6). Similarly, a systemic response of the entire body to high altitude is triggered by hypoxia (7, 8). Hypoxia is an integral part of the pathophysiology of many diseases, including chronic obstructive pulmonary disease (9), heart failure (10, 11), sleep apnea syndrome (12), anemia (13), and cancer, and its basic research can reveal mechanisms that may 1 day be exploited in therapy development (14).

*In vivo* models of hypoxia face considerable shortcomings (15); therefore, cell cultures represent a viable option for this line of research. However, the importance of the precise modulation and definition of hypoxia is often not reflected in the design of *in vitro* experiments, due to historical reasons (16) and technical limitations (17–19). However, with the advancements in various scientific fields, including cell biology and material science, the requirement for adequate control of pericellular oxygen levels in the experimental setup increases in importance, particularly as technological solutions become more readily available.

In this review, we aimed to summarize the current approaches in experimental hypoxia research with special emphasis on cell culture models. The topics covered include the physical limitation of gas diffusion in liquids, methods of inducing sustained and intermittent pericellular hypoxia, and measurements of dissolved oxygen. We also discuss the physiological relevance of mimicking the oxygen dynamics of certain diseases in cell cultures as closely as possible and the implications of the mentioned principles on *in vitro* models mimicking tissue normoxia.

## *In Vivo* Models of Hypoxia

Humans as well as animals can be exposed to hypobaric (HH) or normobaric hypoxia (NH) in order to study wide variety of diseases, including pulmonary hypertension (20), reoxygenation injury (21), pre-eclampsia (22), hypoxic insult of the brain (23), and diabetic retinopathy (24). While HH, which physically resembles a high-altitude environment, is induced by decreasing atmospheric pressure under 101 325 Pa (1 atm, 760 mm hg) typically in a tightly sealed hypobaric chamber (25), NH exposure is based on the reduction of the partial pressure of oxygen (pO_2_) at normal atmospheric pressure, which typically occurs through the administration of nitrogen to a face mask (26), hypoxic tent (27), or environmental chamber (22, 23).

It remains debatable as to whether the two hypoxic states are interchangeable under experimental settings (8). Several differences have been observed by multiple studies, such as in minute ventilation, tidal volume, peripheral O_2_ saturation, arterial CO_2_ pressure, and exhaled NO levels, which appear to be lower and acute mountain sickness symptoms more pronounced in HH However, these symptoms as well as minute ventilation only differ during the acute phase of hypoxia, possibly due to the initial difference between alveolar and ambient N_2_ tension in HH, whereas long-term effects of both states are comparable. Other parameters such as arterial pressure of O_2_ and CO_2_ have been found to be either similar or variable depending on the study (7, 8, 28). Biochemical markers of hypoxia have been measured and found to be equivalent during exercise in NH and HH, with both conditions being different from exercise in a normoxic environment (29).

## Cell Cultures

Ethical problems as formulated by the “3R's” rule, cost-related issues, and limited reproducibility in humans remain the most apparent hurdles of animal model applications (15). Human cell cultures represent a compelling alternative in important areas of biomedical research, such as drug discovery (30) or disease modeling (31), largely due to important advancements in pluripotent stem cell applications over the past two decades (32, 33).

Recent advancements in tissue engineering have enabled researchers to perform *in vitro* experiments not only at the cellular and molecular levels, but also to explore inter-cell and inter-organ interactions using three-dimensional (3D) cell models and complex organoids (34, 35). Nevertheless, significant variability in laboratory-to-laboratory protocols and procedures hamper reproducibility and impose challenges for interpretation and generalizability of results. Despite numerous factors, including cell confluency, composition of culture media, and frequency of media exchange typically reported in method descriptions, a fundamental factor for cell life—pericellular oxygen level—remains largely overlooked. An accumulating body of literature suggests that oxygen levels in standard cell culture experiments not only significantly deviate from a physiological range, but also shows that pericellular oxygen levels vary dramatically under different experimental settings, cell types investigated, cell confluency, and volume and timing of media exchange (17, 18, 36). The key determinants of pericellular oxygen levels and possible means of their control in cell cultures are summarized in the following text.

## Hypoxic Cell Culture Models

### Oxygen Levels in Cell Cultures

In a standard cell culture experiment, cells are kept in incubators that maintain the following stable conditions: temperature of 37°C, atmospheric air (21% volume fraction of O_2_) enriched by 5% CO_2_, and humidity provided by spontaneous water evaporation (37). The volume fraction of oxygen in the incubator atmosphere reaches 18.6% (132.5 mmHg) because of the addition of partial pressures of CO_2_ and water vapor, as described by Dalton's and Amagat's laws (38). Thus, 18.6% O_2_ and its corresponding partial pressure in the incubator is what many would consider as a conventional, standard, or “normoxic” setup (36). Two important limitations of such a paradigm must be addressed. First, pericellular oxygen levels are dramatically different from oxygen levels in the incubator atmosphere, as discussed below. Second, a physiological range of oxygen levels observed in tissues *in vivo* (tissue normoxia or physioxia) is profoundly variable and significantly lower, as discussed in section Hypoxia Mimetics. In fact, physiological alveolar partial pressure falls below that of the incubator oxygen level, following the alveolar gas equation (39).

The first concerns about the possibility of limited pericellular pO_2_ were voiced over a century ago (40), with the first confirmations of pericellular hypoxia reported during the early years of conventional cell culture experiments (17, 41). Metzen et al. (17) showed that under common normoxic conditions as described above, adherent cells may suffer from pericellular hypoxia or even anoxia. When measuring pericellular O_2_ levels 24 h after media exchange, it was found that the cell lines with a high oxygen demand (e.g., human hepatoma Hep3B and HepG2, and renal epithelial LLC-PK_1_ and LLC-MK_2_ cell lines) eventually reached an anoxic state. These authors developed mathematical model based on Fick's law calculating expected pericellular O_2_ levels, which were subsequently verified by real-life measurements.

It has since been acknowledged that in conventional cultureware, the only way for oxygen to reach the adherent cells is by diffusion through the water-based medium overlay. Moreover, if the oxygen consumption rate of cells that exhibit higher metabolic activity exceeds the speed of oxygen delivery (determined by the oxygen solubility coefficient, diffusion constant, medium overlay height, surface area, and partial pressure of oxygen above the medium), the pericellular oxygen pressure eventually equilibrates at a hypoxic or anoxic value after 2 h following medium exchange (17). However, oscillations of pericellular O_2_ tensions around the equilibrated state are also known to occur. These periodical changes likely occur because of a decrease in the respiratory rate following the depletion of oxygen around the cells and, as its availability begins to increase again, oxygen consumption increases as well, exhausting its supply and completing the cycle. It has also been proposed that these oscillations are what ultimately drive the molecular response to hypoxia (36).

Cell surface area and pO_2_ are characterized by the dimensions of the culture dish and the 5% CO_2_ incubator atmosphere. Thus, the medium overlay height represents the main variable that limits oxygen diffusion (17). The medium height and cell oxygen consumption rate both determine pericellular oxygen concentration, and thus significantly affect contemporary cell culture research owing to the lack of standardization (reporting) of media amount supplied to cells and the attention given to the differences in oxygen demand of different cell lines under various experimental conditions (17, 36).

An existing discrepancy in current terminology must be discussed here. Namely, there is a lack of consensus in the usage of terms and units when it comes to measurements of pericellular O_2_ levels. Many researchers describe the pericellular oxygen availability as concentration given in percent (18, 19, 42–48). However, this is not accurate from the physical point of view as these studies are actually referring to the volume fraction of oxygen in the ambient air that corresponds to the actual molar concentration of oxygen in an aqueous solution—the medium. Based on Henry's and Amagat's laws, the molar concentration is determined by the volume fraction of oxygen in the air and the atmospheric pressure, which can be summarized as the pO_2_ (38, 49, 50). Therefore, should the atmospheric pressure decrease with an increasing altitude, the actual amount of oxygen in the cell culture medium would also change accordingly.

Such disparities affect not only standard cell culture where no attention is given to the pericellular pO_2_, but they also make it difficult to unify the results of published material on *in vitro* hypoxia measurements. We could hypothesize that authors using the term oxygen concentration refer to atmospheric pressure at sea level; however, as they usually do not elaborate sufficiently enough on how they derived their concentration values from pericellular pO_2_ measurements, we cannot be certain of this. Therefore, we are unable to precisely calculate pO_2_ and/or molar concentrations of these experiments and directly compare them to the units used in other studies.

Therefore, we propose that this terminology should not be used in future to avoid further confusion. Instead, the values of molar concentration of oxygen (51) or oxygen partial pressure, which can easily be converted to one another under constant temperature following the ideal gas equation (36) or Henry's law (49, 50), should be used henceforth to promote reproducibility and intelligibility of the results. In fact, O_2_ partial pressure is also a non-sensical term to use when describing the concentration of a gas in a liquid, which only corresponds to partial pressure of a gaseous phase (49, 50). However, it has become the most widespread way of characterizing oxygenation in both medical physiology and clinical practice because the majority of oxygen in the blood is not dissolved, but is transported while bound to hemoglobin (50).

### Pericellular Oxygen Measurements

**Figure 1:**
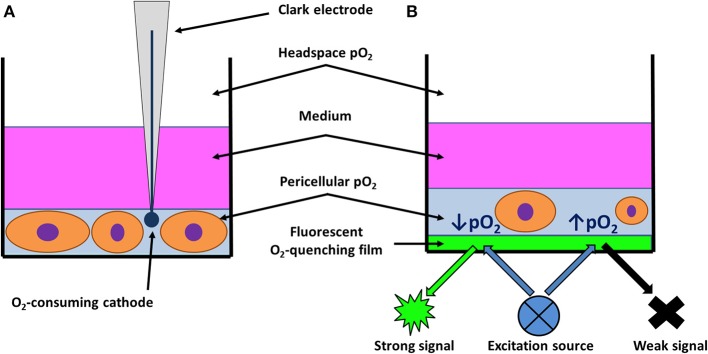
Schematic drawing of pericellular pO_2_ measurement. **(A)** Clark electrode immersed in media, enabling measurement of pO_2_ at any depth including pericellular area of adherent culture. Adapted with permission according to (18). **(B)** Optical pO_2_ sensor based on quenching of fluorescence by oxygen may be positioned as a thin film on the bottom of cultureware surface. To perform measurement, a fluorescent excitation source is placed below the cultureware. Strong emission signal is registered from areas with low pO2, while oxygen-mediated quenching results in limited emission from oxygen-rich regions. Adapted with permission according to (53).

Before moving on to removing the issue represented by limited oxygen supply to cells *in vitro*, one must first be able to characterize the oxygenation of the cells properly. One way of doing this is using a polarographic O_2_-sensitive electrode, named the Clark electrode after its discoverer (52). Based on the principle of electrolytical reduction of oxygen, this electrode allows for the measurement of oxygen levels at a precise location in a pericellular area. However, manipulating the electrode can also disrupt the cells/samples. Due to its construction, it does not enable for simultaneous detection of O_2_ levels in all dimensions, which prevents it from measuring the concentration gradients in 3D cultures or inhomogeneities in the microenvironment of the cells (18). Furthermore, the electrode itself has non-negligible O_2_ consumption, which must be considered during prolonged experiments, as it requires re-calibration or stirring of the medium (Figure 1A) (4, 17, 52). To overcome these limitations, alternative and complementary methods have been developed to monitor pericellular O_2_ levels.

Certain tissue dyes such as Hypoxyprobe (pimonidazole hydrochloride) coupled with monoclonal antibodies are provided only with semi-quantitative assessments of hypoxia (4). Moreover, Hypoxyprobe is generally designed for use in patients and animal models to observe hypoxia of an explanted tissue (54, 55), although sporadic use *in vitro* has also been reported (56). Nevertheless, there is another staining method that yields exact values of pO_2_, which is based on oxygen-mediated quenching of the fluorescent signal that is inversely proportionate to pO_2_ (57), the intensity of which is quantifiable by a microscope (58) or commercially available devices (59). This principle can also be implemented using dishes with pre-calibrated oxygen sensors positioned at the bottom of each well (Figure 1B) (46, 53). Custom-made amperometric electrodes, which utilize ion current quantification to assess analyte concentration, may also be integrated to the cell culture system in a similar fashion (47).

### Sustained Hypoxia—Diffusion Challenge

Motivated by the ability to monitor pericellular pO_2_, as demonstrated above, investigators developed multiple approaches that enabled O_2_ control at the cell level throughout experiments. The most straightforward option was the empirical adjustment of air composition in the incubator based on the measurement of oxygen tension around the cells, which has been used repeatedly owing to its technical ease (44, 48, 60, 61). However, it has been demonstrated that in metabolically active cells (17), pericellular O_2_ reaches extremely low levels owing to a mismatch between cellular oxygen consumption and the amount of O_2_ delivery by diffusion through the culture media. Early attempts to tackle the limitation of diffusion were rather simple and aimed to drastically reduce the height of the medium above the cells. Unfortunately, the amount of medium is crucial for keeping the cells well-provisioned with nutrients and for maintaining a stable environment. Hence, this method is suboptimal for prolonged cell culture. Additionally, the meniscus of the medium forming within the culture well-causes a significant difference in the diffusion distance across the culture surface, which requires maintaining the cells in the middle of the well (62), which is difficult to achieve when using cell lines with significant proliferative capacity. Alternatively, researchers used stirring or shaking of the culture vessel as a simple method to increase oxygen diffusion (36), although this was at the cost of inducing mechanical stress to the cells (63).

To overcome the above-mentioned limitations, placement of a commercially available culture dish with a gas-permeable bottom made of a fluorocarbon membrane in a modular incubator chamber (an air-tight sealed plastic chamber) filled with atmosphere-containing predetermined O_2_ and CO_2_ levels has been employed (45, 64). Using this setup, adherent cells receive O_2_ directly from the modular incubator chamber atmosphere via the permeable membrane without having to rely on diffusion through the medium, which has been shown to be both effective and simple in terms of being able to regulate pericellular pO_2_ closely with relatively fast equilibration times (45, 65). Additional advantages of modular incubator chambers compared to standard incubators (with or without control of O_2_ levels) include the elimination of gas leaks (changing oxygen levels) and the minimization of convective forces associated with incubator openings (66).

### Intermittent Hypoxia—Equilibration Challenge

While reaching and maintaining sustained pericellular O_2_ levels *in vitro* is feasible via the methods described in the previous section, a much greater challenge lies in developing a system that provides researchers with a means of modeling IH, where precise cyclic control of pericellular oxygen tension as well as its fast equilibration is mandatory. OSA syndrome represents one of the most blatant examples because the cycles of IH occur as often as 60 times per hour (67). This dictates the need to create a system in which pericellular pO_2_ would change every minute, while achieving equilibrium with the gas phase during each period.

Mere fast-paced changes (within minutes) in headspace O_2_ levels inside a modular incubator chamber cannot achieve the desired pericellular pO_2_ as the equilibration of oxygen levels across media takes a significantly longer time, depending on the thickness of the medium overlay. For instance, a 170 μm-thick media barrier between a human osteosarcoma cell line and the incubator atmosphere allows for a 1.5 min long equilibration, whereas this time increases 10-fold if the overlay height is a mere 1 mm (68). Perforation of a culture plate lid has been shown to speed up the equilibration between the pericellular and headspace pO_2_, suggesting that this barrier is also important in slowing down the diffusion of O_2_ toward adherent cells (19).

#### Flow-Through Systems and Microfluidics

**Figure 2:**
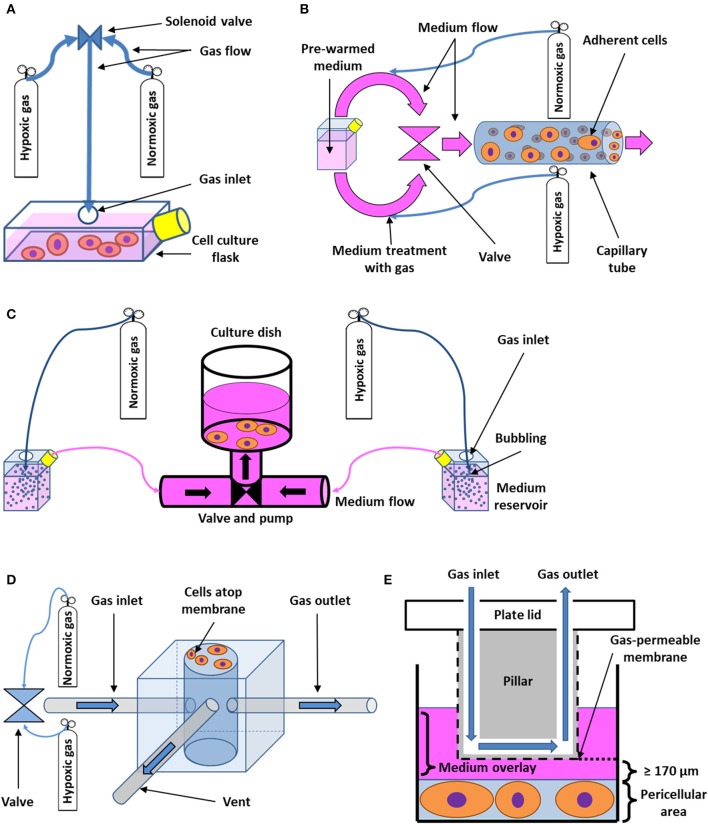
Schematic drawing of flow-through systems for *in vitro* intermittent hypoxia. **(A)** Administration of desired gas mixtures directly into a cell culture flask. Cyclic change of hypoxic and normoxic atmosphere is ensured by a solenoid valve. Adapted with permission according to (69). **(B)** Perfusion-based system with cells growing on the wall of a capillary tube. Pre-warmed medium is first divided into two circuits, which are treated with both hypoxic and normoxic gas mixtures, respectively. Both circuits then alternately open into the capillary area seeded with cells by passing through a valve. Adapted with permission according to (70). **(C)** Bioreactor based on cyclic perfusion of cells with hypoxic and normoxic medium prepared by bubbling with gas. Both hypoxic and normoxic circuit has its own pump, pushing the medium in and out of a culture dish through a periodically-opening valve. Adapted with permission according to (71). **(D)** Microchip for intermittent hypoxia coupled with cyclic stretch to cell cultures. Varying gas mixtures are pushed into a well at the center of the microchip via a valve or a gas blender, flowing out of the well-through a separate gas outlet tube. A venting tube connected to the well-leads to a solenoid valve (not shown), which serves the purpose of periodically changing pressure inside the well, applying indirect mechanical stimuli to cells growing on the outside of a gas-permeable membrane of the well. Adapted according to (63) under the CC BY license. **(E)** Cell culture insert for intermittent hypoxia. A pillar fixated on the lid of a cell culture plate with integrated channel for gas perfusion is immersed in medium in order to reach close vicinity of pericellular area. The desired gas is then delivered to cells via the channel and a gas-permeable membrane as the thickness of the diffusion barrier represented by the medium may be limited down to 170 μm. Adapted with permission according to (68).

Multiple models for IH *in vitro* that meet the requirements of a more dynamic approach have been developed and validated. For instance, cyclic changes of gas mixtures flowing directly into cell culture flasks (Figure 2A) (69) or microfluidic devices capable of directly supplying cells with precise gas mixtures via a system of miniature channels (72–74) have been used. Other perfusion-based systems rely on growing the cells directly on the walls of tube-like channels, through which medium is pushed (Figure 2B) (70). Alternatively, bioreactors containing peristaltic pumps that drive the hypoxic and normoxic media to the cells in a cyclical manner from reservoirs pretreated by bubbling the desired gas mixtures through the liquid may be employed (Figure 2C) (71, 75). A shared downside of perfusion-based approaches is the shear stress being exerted on the cells during perfusion with the equilibrated medium and/or gases. To minimize this problem, a polydimethylsiloxane (PDMS) microfluidic chip consisting of a chamber through which oxygen-rich and oxygen-poor air is pumped in a periodic fashion has been constructed. Unlike in other microfluidic settings, cells are not in the chamber itself but rather are in direct contact with it by growing on a gas-permeable membrane, which allows for close control and fast equilibration of pericellular O_2_, while diminishing mechanical stress. Furthermore, the modulation of pressure in the chamber mediates a cyclical stretching of the cell culture that simulates the periodic expansion of the heart or lung (Figure 2D) (63). Using a similar principle, the development of a PDMS pillar with an oxygen perfusion channel, coated with Parylene-C to ensure better oxygen isolation and faster equilibration, has been reported. The pillar can be mounted on the top of each well of a common culture dish (Figure 2E) (58, 68). It then streamlines a precisely defined gas mixture in very close vicinity of the cellular monolayer, bypassing most of the culture medium diffusion barrier. Cells are isolated from the apparatus itself; therefore, no shear force is present (58). Unlike the microfluid approach (63), this method is well-suited for culture plates using standard-sized wells as it does not have to cope with the limited space of miniaturized chambers inside a chip.

#### Membrane-Bottom Based Approaches

**Figure 3:**
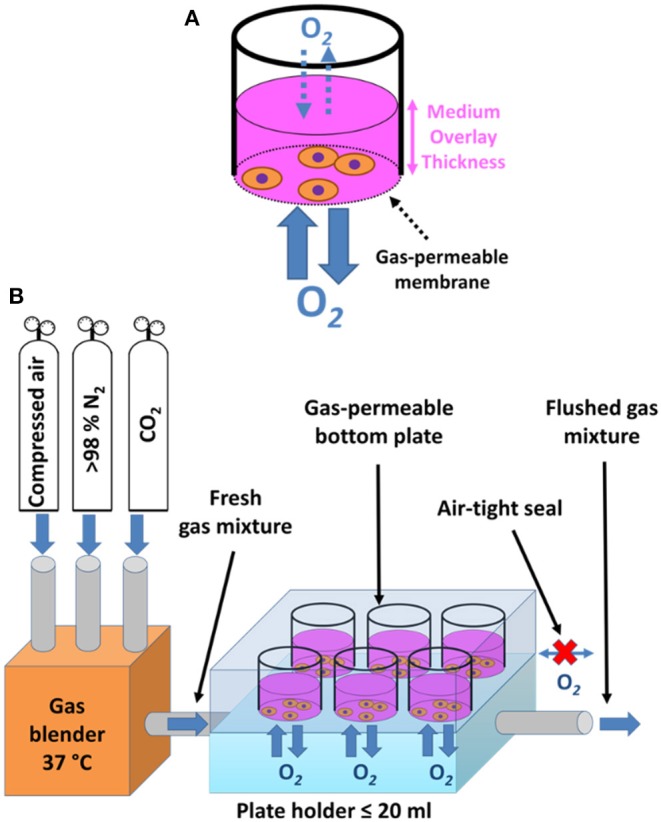
Schematic drawing of membrane-bottom systems for *in vitro* intermittent hypoxia. **(A)** Periodic change of pO_2_ in a cell culture cabinet is appropriately reflected only in the pericellular area of a gas-permeable membrane cultureware, which allows for unlimited diffusion of oxygen and fast equilibration times, whereas standard plastic dishes only enable diffusion through the medium overlay, posing as a barrier. Adapted with permission according to (45). **(B)** Custom-made plateholders of a volume no more than 20 ml connected to gas-permeable bottom dishes in an air tight fashion serve the purpose of limiting the volume of gas mixtures that need to be periodically exchanged during intermittent hypoxia regimes. The required gas mixtures are prepared and pre-warmed in a gas blender, brought into the plate holder by gas inlet tubing, allowing for unlimited diffusion of oxygen between the plate holder and adherent cells, and subsequently flushed out through a gas outlet tube to make room for a fresh gas mixture. Adapted with permission according to (64).

Growing cells on commercially available cultureware dishes, fitted with gas-permeable fluorocarbon membrane, may be used not only for maintenance of sustained hypoxia (as mentioned above), but also for efficient recreation of intermittent hypoxia. Enclosing such a culture dish in a sealed cabinet while controlling O_2_ levels, e.g., by a programmable digital controller, enables for rapid and reproducible exposure of cells to intermittent hypoxia, without the need for diffusion through a culture medium (Figure 3A) (45, 65). The limitations of such an approach include the large volume of gases (O_2_, N_2_, and CO_2_) required to achieve the rapid exchange of the inner cabinet atmosphere associated with significant culture media evaporation (despite humidification) and gas pressure changes inside the cabinet or inside the sealed culture dishes due to heat expansion of gas (45), all of which adversely affect the performance of IH exposure. Minoves et al. (64) modified the setup by combining the gas permeable culture dishes with a customized plate-holder equipped with its own gas tubing. This was designed to seal the plate off from the surrounding atmosphere, replacing the hypoxic chamber with a significantly smaller space, and thus limiting the volume of air to be pumped in and out during each cycle (Figure 3B).

### Hypoxia Mimetics

Instead of exercising control over oxygen availability, some *in vitro* as well as *in vivo* models utilize hypoxia mimetic agents which simulate hypoxic conditions predominantly by increasing availability of intrinsic HIF-1α in standard cell culture settings. This methodology can be used for both sustained and intermittent hypoxia models, the latter of which can be achieved by cyclic exposure to the agent (76, 77).

Precise mechanism of action of hypoxia mimetic agents may vary depending on the particular agent used. Many of the compounds inhibit HIF-prolyl hydroxylases (PHDs), which are crucial for HIF-1α degradation. Cobalt chloride (CoCl_2_), arguably the most widely used hypoxia mimetic, competes with Fe^2+^ ions, which are necessary for enzymatic activity of PHDs. Iron chelators, such as deferoxamine mesylate (DFO) work by similar means. Dimethyloxalylglycine (DMOG) is a 2-oxoglutarate analog, which also inhibits PHDs and may be utilized in hypoxic cell culture models (78–81). However, one of the downsides to the most common PHD inhibitors is their cytotoxicity. To overcome this problem, another PHD inhibitor hydralazine has been successfully employed to mimic hypoxic conditions and proved to be significantly less cytotoxic than CoCl_2_ (82).

Nevertheless, other mechanisms of action, such as mitochondrial uncoupling in case of bafilomycin A1 have been exploited in cell culture models of hypoxia (83). Inhibition of proteasome degradation, miRNA approaches and application of isoflurane or N-acetyl cysteine also have HIF-1α stabilizing effects, but these methods are predominantly utilized to ameliorate ischemia-reperfusion injury rather than to mimic hypoxia (80, 81).

### Role of Tissue Normoxia in Hypoxic Models

To realize the importance of tissue normoxia for hypoxic cellular models and its distinction from hypoxia, one must first understand that the use and definition of the terms “normoxia,” “hypoxia,” and “hyperoxia” are somewhat arbitrary in cell culture literature as the composition of headspace gas and not the actual pericellular microenvironment is typically considered. Hypoxia is usually defined as the insufficient supply of oxygen to the relevant tissue, although several other definitions have been proposed. These definitions revolve around the state of mitochondrial respiration and temporal dynamics of the molecular apparatus that are centered around HIF-1α (84).

Different types of tissues, however, have various oxygen demands (85) and variable capillary network and blood flow regulation, resulting in largely different tissue pO_2_ *in vivo*. Varying pO_2_ in different body organs in humans have been comprehensively reviewed by others (4). The unique pO_2_ of each organ, called physioxia or tissue normoxia, warrants more elaborate experimental settings, ideally mimicking such tissue-specific physioxia *in vitro*. Clearly, considering the usual environment of an incubator as “normoxic” represents a failure to recapitulate basic physiological parameters. In fact, a standard incubator atmosphere (18.6% O_2_) might induce severely “hyperoxic” conditions in some cultured cells and nearly anoxic pericellular oxygen levels in other cultured cells—all fundamentally deviating from physiological oxygen levels observed in tissues (4, 84). Any particular *in vitro* model of hypoxia should thus aim to reach oxygen levels lower than tissue normoxia (physioxia). Ideally, oxygen tensions present in the tissue or disease *in vivo* should be implemented if the exact values are known.

For example, pO_2_ in human adipose tissue has been found to be approximately 55 mmHg, but lower pO_2_ levels have been measured in the subcutaneous fat of obese subjects with a possible link to inflammation of the tissue (86), and thus also the pathophysiology of type 2 diabetes mellitus (87). It has also been reported that adipose tissue-derived stromal cells retain their natural phenotype when the O_2_ levels of their physiological niche are maintained (88). Similarly, pO_2_ in fetal arterial circulation, as opposed to adults, equals approximately 30 mmHg (89), whereas that of a trophoblast is slightly higher, 40–60 mmHg (48). The ideal pO_2_ for early stage human embryonic development presented *in vivo* and utilized by *in vitro* fertilization laboratories appears to be in the range from 2% (~15 mmHg) to 5% (~38 mmHg) tension of oxygen (90). This knowledge has been exploited in studies of embryonic and induced pluripotent stem cells as the quality of pluripotent stem cell culture characterized by proliferative capacity and expression of pluripotency markers is significantly improved when the cells are grown in incubator atmosphere commonly described in literature as hypoxic, ranging from 1 to 10%, while higher O_2_ concentrations referred to as normoxic showed to be detrimental (91–98).

Finally, tissue pO_2_ has been measured and found to be significantly reduced in the majority of tumors in patients. Knowing these values is particularly important because the properties of cancer cells, such as sensitivity to chemotherapeutic agents, change dramatically under hypoxic conditions (4). Modified tissue pO_2_ has also been observed and recorded in myocardial infarction (99), retinopathy (100), and pre-eclampsia (101), and to a limited extent also in OSA (102, 103).

## Effects of Pericellular Po_2_ Control on Hypoxia Signaling *in vitro*

The need to tailor experimental conditions of *in vitro* hypoxia, namely O_2_ levels, to meet those found in living patients is further purported by mechanisms occurring in hypoxia at cellular and molecular levels, many of which are directly involved in disease pathophysiology. These predominantly include the upregulation of HIF-1α (104), nuclear factor-kappa B (NF-κB) (105, 106), and reactive oxygen species (ROS); the decreased availability of nitric oxide (107); or complex changes in ion channel activity (108). It has been shown multiple times in both cell culture and *in vivo* that these processes are very tightly governed by O_2_ concentration (109–112). Namely, HIF-1 expression, which is a central molecule in cellular signaling during hypoxia, increases exponentially as oxygen tension decreases (113). Using gas-permeable plates that ensure close control of pericellular pO_2_ cycles for IH has found that different pO_2_ levels around the cells and its dynamics significantly vary in their effect on HIF and NF-κB expression (45). This would explain the contradictory results reported by different studies employing varying modes of hypoxia induction when exploring its influence on HIF mechanisms (69, 75).

### Sustained Hypoxia

The influence of pericellular pO_2_ measurement and control can be shown by the example of tumor hypoxia. The *in vivo* tumor microenvironment is characterized by unique oxygen tension values, which may have a considerable influence on clinical treatment, influencing the efficacy of anti-cancer drugs and radiotherapy (4). In fact, *in vitro* hypoxia has already been utilized to simulate the effect of oxygenation dynamics on breast cancer radiosensitivity, which was found to be diminished in hypoxia, taking advantage of the ability to continuously measure pericellular pO_2_ (114). The cancer cell culture model has also been employed to develop a new hypoxic probe, which accumulated inside tumors *in vivo* as well as *in vitro*, which implies that this culture condition might prove to be a useful tool in drug testing (115). To this end, a microfluidic chip, capable of creating pO_2_ gradients and evoking multiple oxygenation states, has been developed (116). A myriad of other microfluidic devices could also be devised for the purpose of accurately recreating tumor phenotypes in a dish (63, 117).

*In vitro* hypoxia may also be studied to uncover the molecular mechanisms ameliorating ischemia/reperfusion injury in neurons (118) or cardiomyocytes (119). A perfusion-based model of murine cardiomyocytes subjected to abrupt anoxia and reperfusion was discovered to be an optimal platform for demonstrating the opening of mitochondrial permeability transition pores (mPTP). Because mPTP is a protein complex in mitochondria activated during ischemia-reperfusion injury leading to cell death, this model could be utilized to study its molecular nature, which still has not been fully elucidated, and eventually to develop a pharmacological approach to block it (119). In addition, a steady perfusion-based microfluidic system has been developed to continuously monitor the effects of hypoxic insults on the electrophysiological properties of cardiomyocytes. At the hypoxic level, which is translatable to a 5% oxygen concentration, L-type calcium currents were decreased that corresponded to *in vivo* observations and the stunned myocardium hypothesis (120).

### Intermittent Hypoxia

The significance of precise pO_2_ maintenance also applies to IH modeling. A recent study explored the effect of IH in OSA on insulin resistance and the results from the *in vitro* model, which utilized gas-permeable dishes and OSA pathophysiology, were in accord with the animal model and patient cohort observations, including changes in NF-κB modulation (65). Moreover, adipocytes grown on the same type of cultureware and that were subjected to clinically-relevant IH exhibited an accumulation of triglycerides, which correlates with the observed link between obesity and OSA in patients (42). Conversely, culturing adipocytes in suboptimal settings without pericellular pO_2_ monitoring has led to conflicting results on whether hypoxia increases HIF expression (87, 121, 122).

Similar to adipocytes, when other cell types underwent a protocol of IH with defined pO_2_ *in vitro*, the results were consistent with other IH models. For instance, gene expression profiles of neutrophils, monocytes, and airway epithelial cells all matched the results found in OSA patients, further hinting at the role of inflammation in the pathophysiology of the disease (123–126). Constant monitoring of pO_2_ of the PC12 cell line confirmed the central role of HIF-1α in the molecular response to IH (127). The same molecule was found to be upregulated in skin vasculature taken from the biopsies of OSA patients as well as in the aortas of mice and human cultures of coronary artery endothelial cells, where IH was maintained by gas bubbling in the medium (128). Additionally, a protective mechanism of pancreatic cells exposed to IH and hyperglycemia based on ROS reduction both *in vivo* and *in vitro* has been described (129).

Furthermore, a myocardial ischemia model was adopted using gas-permeable culture dishes to study the effect of different hypoxic modalities. Continuous measurements of pericellular pO_2_ showed that IH, simulating repeating cycles of ischemia and reperfusion, OSA, or several pulmonary conditions resulted in a considerably more pronounced inflammatory response and cell injury than that of mild hypoxia, comparable to or at earlier stages even greater than that of severe hypoxia. This is in agreeance with the fact that OSA is considered to be an independent risk factor of cardiovascular disorders (130). The addition of cyclic stretching mimicking heart and/or lung movements was demonstrated to act synergistically with IH, upregulating the HIF-1α pathway in mesenchymal stem cells, and showing that this model could be superior to others when simulating IH in these organs (63).

### Downsides of Conventional Systems

Discrepancies in the results of the role that hypoxia has on cell cultures could be attributed to a number of variables, such as using different cell types (69) or species (87). Nevertheless, if the native pO_2_ of the tissue type in question as well as its temporal development in the disease being studied were to be respected and incorporated into the *in vitro* model as suggested (4), variability in results could arguably be reduced.

Despite this, many recent publications pertaining to *in vitro* hypoxia still implement the simplified model, which does not consider the difference between headspace and pericellular O_2_ tensions. Such studies encompass various areas of hypoxic research, ranging from IH (131–133) to the tumor microenvironment (134, 135) to reperfusion injury modeling. In regards to the latter, placing cells into anoxic conditions generally reflects the ischemic insult occurring *in vivo* (132, 136). Notwithstanding, the absence of pericellular O_2_ measurements makes it virtually impossible to ascertain whether there is a higher concentration of oxygen in the control group and by what margin. This makes any interpretation of the results complicated, especially because the cell cultures used in these studies for their sensitivity to ischemic hypoxic injury, such as cardiomyocytes (132), neurons (137), kidney (138), and endothelial cells (139), tend to have relatively high oxygen consumption rates (85). Moreover, these studies generally do not include information about the height of the medium overlay above the adherent cell culture, which introduces yet another unknown variable that possibly affects the results and reproducibility (17, 18). Furthermore, certain studies employed only chemical insults to mimic hypoxia (134, 140–142). Logically, some studies then report that cell cultures have no merit in this area of hypoxia research (139), while others state that the results gained from these models are in line with studies *in vivo* (136, 143). This highlights the importance of maintaining strict conditions for *in vitro* hypoxia characterizing the experimental setup in detail, including pericellular pO_2_ values and, in case of IH, pO_2_ equilibration time.

## 3D Cell Cultures

A special consideration must be given to 3D tissue cultures as they differ significantly from the cultured adherent cells discussed in this review. Introducing the element of three-dimensionality to cell cultures, which is arguably an intrinsic feature of all multicellular organisms, can improve the potential of *in vitro* models to recapitulate the *in vivo* environment (144). This also applies to conditions in which hypoxia plays an important role, including cancer, as the 3D organization has been found to play an integral part in tumor biology, considering the actual tumor architecture and dynamic interactions with the surrounding environment (145). While some models employ only single cell type spheroids (146), more complex platforms, reflecting physiological interactions found *in vivo* include multiple cell types in a 3D structure, such as cancer stromal or endothelial cells (147, 148) as reviewed earlier (149). For example, a study investigating the role of OSA in cancer employed a 3D cell culture model comprising both tumor spheroids and patient-derived monocytes subjected to IH and found that the monocyte-induced HIF-1α-dependent production of VEGF promoted tumor growth (150), providing some molecular insights into the link between the two diseases (151).

Similarly, 3D cell culture technology has been used to study the effect of hypoxia in the context of ischemia in various cell types, including cardiomyocytes (152–154), astrocytes (155), endothelial cells (156), and hypoxia related to pulmonary fibrosis in fetal lung fibroblasts (157). Furthermore, the effects of both continuous hypoxia and IH on vascular sprouting has been explored in endothelial cells (158–160). Hypoxic 3D tissue structures comprising retinal astrocytes and endothelial cells represent a useful drug-screening tool, outperforming standard 2D co-cultures (161).

**Figure 4:**
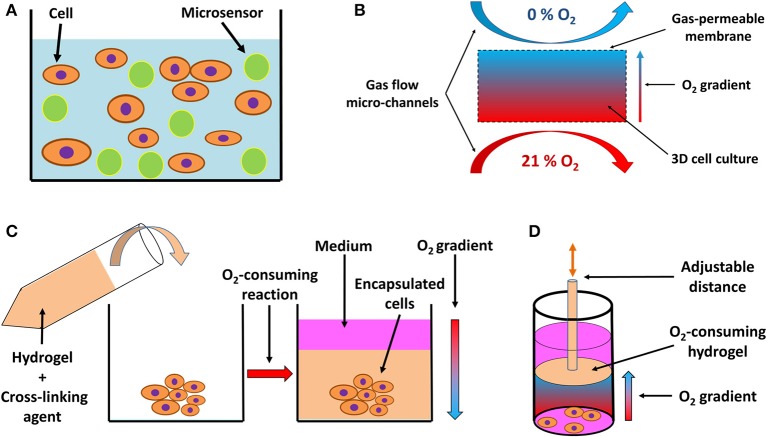
Schematic drawing of systems for 3D *in vitro* hypoxia. **(A)** Fluorescent sensor-laden microbeads incorporated into a 3D hydrogel with cells, serving as a pO_2_ reporter throughout the 3D system. Based on a drawing by (168). **(B)** 3D culture positioned between two gas perfusion microchannels, which are in contact with the culture via a gas-permeable membrane, allowing for an oxygen gradient to be formed across the culture between an oxygen-rich and oxygen-poor environment. Adapted with permission according to (169). **(C)** Encapsulation of a 3D cell culture or explanted tissue by an oxygen-consuming hydrogel, creating a hypoxic environment. The oxygen consumption is a result of the cross-linking reaction and hydrogel formation, resulting in an oxygen gradient from the top layer of the culture to deeper, more oxygen-deprived regions. Adapted with permission according to (165). **(D)** Preformed oxygen-consuming hydrogel immersed in a 3D culture creates a hypoxic environment with an oxygen gradient toward the hydrogel. The gradient and the level of hypoxia can be adjusted by moving the hydrogel across the culture on a mobile pillar. Adapted according to (170) under the CC BY license.

With the proliferation of experiments conducted in 3D cell cultures, critical consideration of pericellular pO_2_ is warranted, particularly because the element of three-dimensionality and variable thickness of cellular structures introduces additional irregularities that hamper gas diffusion and lead to the formation of oxygen concentration gradients (162, 163). Several novel approaches and techniques have emerged tackling the challenges of pO_2_ in 3D tissue structures. Analogically to adherent cell cultures, oxygen-sensing microelectrodes have been employed to measure pericellular oxygen gradients in thicker hydrogel-based tissues (164, 165). However, the disadvantages of this approach, such as its invasive nature, time demands and technical challenges requiring repetitive calibrations and measurements in different spots inside the tissue construct, motivated the search for alternative approaches. A number of fluorescence quenching probes has been tested, which penetrate through cells (166, 167) or are incorporated into microbeads dispersed in a 3D hydrogel (Figure 4A) (168), and subsequently visualized using confocal microscope imaging. Such applications enable the establishment of a dense network of pO_2_ reporter points throughout the 3D cell culture block. Semi-quantitative approaches to the assessment of pericellular pO_2_ in 3D cultures include mathematical models (162) and probes (e.g., Hypoxyprobe) (163) or the incorporation of paramagnetic particles into cellular spheroids with subsequent electron paramagnetic resonance-based detection (171).

A unique feature of 3D cell culture systems is represented by the possibility of actively inducing a controlled oxygen gradient across the model, based on the experimental needs. Such gradients can be induced by perfusion with an oxygen scavenger in the medium (159); by positioning the culture between two micro-channel circuits perfused with gas, each with a different oxygen level (Figure 4B) (169, 172); or by incorporation of an oxygen-consuming reaction of specific hydrogel materials, either encapsulating (Figure 4C) (164, 165) or being in close vicinity of the cells (Figure 4D) (170), and thus regulating the pericellular oxygen levels.

## Conclusion

Cell culture models represent an invaluable research tool for understanding the fundamental mechanisms of the pathogenesis of hypoxia-associated conditions and diseases, as well as for the development of therapies combatting them. However, physical laws pertaining to gas diffusion and oxygen distribution in cell cultures impede pericellular oxygen levels, and thus determine cellular processes. Multiple factors, including media thickness, media mixing, convective forces, cellular oxygen consumption, and headspace pO_2_ determine the pericellular concentration of O_2_, which is significantly different from the O_2_ levels in the gas phase in standard incubators. As even a small change in pericellular O_2_ levels may elicit variable molecular responses, the precise control of pericellular O_2_ levels is required for the appropriate interpretation of the physiological relevance of observed results as well as for laboratory-to-laboratory uniformity. Recent advances have produced several accessible, cost-effective, and high-throughput tools that are capable of emulating constant hypoxic or IH exposure closely reminiscent of the *in vivo* conditions. Moreover, the incorporation of 3D tissues into cellular models of hypoxia might bolster this line of research even further.

## Author Contributions

JPa and JPo conceived and wrote the manuscript and performed the research of bibliography. JPa conceived and made the figures. Both authors directly and substantially contributed to the work and gave permission for this manuscript to be published.

### Conflict of Interest

The authors declare that the research was conducted in the absence of any commercial or financial relationships that could be construed as a potential conflict of interest.
